# Supplementary material for: Rapid turnover of life-cycle-related genes in the brown algae
Source: Genome Biol. 2019 Feb 14;20:35. doi: 10.1186/s13059-019-1630-6 (PMC6374913; doi:10.1186/s13059-019-1630-6)
Supplement: Supplementary file 3 — Protein alignments, corresponding Gblocks html file and CDS sequences for the lineage conserved gametophyte-biased genes. (ZIP 2109 kb) [file 13059_2019_1630_MOESM3_ESM.zip › Con_GA_alignments/Ec-12_003390.aa_ali.fasta-gb.htm]

Ec-12\_003390.aa.fasta.trans.aa\_ali.fasta


## Gblocks 0.91b Results

Processed file: **Ec-12\_003390.aa.fasta.trans.aa\_ali.fasta**  
Number of sequences: **2**  
Alignment assumed to be: **Protein**  
New number of positions: **525** (selected positions are underlined in blue)

```
                         10        20        30        40        50        60
                 =========+=========+=========+=========+=========+=========+
Ec-12_003390     ------------------------------------------------------------
MSTRG.18017.1    MIRVHGSEYDVEASRNVDRATQALVPACSPGALPTDPKHLNAMFVFCTNNWRTYSGDKPT
                                                                             


                         70        80        90       100       110       120
                 =========+=========+=========+=========+=========+=========+
Ec-12_003390     ---------------------------MEKNLKEI-------------------------
MSTRG.18017.1    GEKLARINAVHKQATIGDCTDVEWATPFEKTVLRIWRSYRGTAVLTAKRRFLTLIRDVDP
                                                                             


                        130       140       150       160       170       180
                 =========+=========+=========+=========+=========+=========+
Ec-12_003390     --------------------------CCNTKKGCPRPLMDKHGHNLEQELDTNESLSEFA
MSTRG.18017.1    RLLQIKAYQFLPWGFPTTAEGGRICPYSNAKKGCPRPLMDKYGHNLEQELENNESLADFT
                                             ################################


                        190       200       210       220       230       240
                 =========+=========+=========+=========+=========+=========+
Ec-12_003390     TLKTWLDEVVAQQRCALGLHTPISAEQGAKFSTFFARPECGGFRQHTSGKIHNMVKKVLT
MSTRG.18017.1    NLKGWLDEVAAQQRCALGQHTAISAEMGRTFSTFFARPECDGFQPYTSTKIHNMVKRVLT
                 ############################################################


                        250       260       270       280       290       300
                 =========+=========+=========+=========+=========+=========+
Ec-12_003390     KQFLALLDMQNNPLNFSRVAQIRQRDRVSGLQQYYTSLTGHEFSNEVPCSRDSHHCDTRR
MSTRG.18017.1    KQLLTLVEMQNKPRNFSKIAQSVQRDRVLGLQQYYTNLTGHEFSNEVPCSRDSHHCNTRR
                 ############################################################


                        310       320       330       340       350       360
                 =========+=========+=========+=========+=========+=========+
Ec-12_003390     LAEGGNHTHPVVIRTPPTDLSLYDALIQVRRECVRLGVVPATGPVHSIDHRCRLLQMKVA
MSTRG.18017.1    LAEGSNHTHPVVIRTPPADLSLYDSLIQVRRECVRLGAVPVTGPVDSIDHRCRLLQMKVV
                 ############################################################


                        370       380       390       400       410       420
                 =========+=========+=========+=========+=========+=========+
Ec-12_003390     DFHRKQMLIYESQRRQQWRVDIAPHDAALLKTFSHRHRSEHLRHALDAKDMDRVCVVLES
MSTRG.18017.1    DFHRKQMLVYESQRRQQWRKDIGPHDAAMLKAFSHDHRSDCLRLAMEANDMDRTCLVVES
                 ############################################################


                        430       440       450       460       470       480
                 =========+=========+=========+=========+=========+=========+
Ec-12_003390     GADPSTELRSGLFPMMAAVVKRSIPVIQRLLAAGADINHSNSKGMTALMWAVKRNDYAMV
MSTRG.18017.1    GGDPGTELRSGLFPMMAAVLNRSIPHIKRLRAAGAEIDYANSKGMTALMWAVKRDDYAMV
                 ############################################################


                        490       500       510       520       530       540
                 =========+=========+=========+=========+=========+=========+
Ec-12_003390     DALLEEGADVEVEGCSGWTAMSIASRHGRRDIAQFLVETLRADKVVGDMKATRALNHRSS
MSTRG.18017.1    DSLLDEQADVQVEGRTGWTAMSIASRHGREEIARLLVETLRRDKISGEMKAGRVLNHRST
                 ############################################################


                        550       560       570       580       590       600
                 =========+=========+=========+=========+=========+=========+
Ec-12_003390     VNRGLTPLAIAAIHRNEAMARCLMRLGAKPAVKCHQGHVAGEHAAKAGWGVFGLWLQETQ
MSTRG.18017.1    VNGGLTPVGIAAIHRNEAMARFLMRLGAEPGVRCHQGHVAGEHATKAGWSVFGLWLQETQ
                 ############################################################


                        610       620       630       640       650       660
                 =========+=========+=========+=========+=========+=========+
Ec-12_003390     AFGDRGVYTFADMNAENALRVAEQRMLDAISSGKTVQDDSKRNSRTTSVASNSGGSRPGP
MSTRG.18017.1    AYGDSGVYTFADMSAESALRIAAVRMLDAISSGATVEDGMKKESRPSSAANDN---RPRS
                 ##################################################          


                        670       680       690       700       710       720
                 =========+=========+=========+=========+=========+=========+
Ec-12_003390     AMASSSGRQVSSATSGDTRPLSSASSRRRPTSSRKQGEPRPTPAIPATATEEEHNQTLPE
MSTRG.18017.1    GV--ASGNLVSPALSGRT-VV---------------------------------------
                      #############                                          


                        730       740       750       760       770       780
                 =========+=========+=========+=========+=========+=========+
Ec-12_003390     PLPGVPSESARDGDLSEFLDVTQFQLGQIRSKVLLTVSILHEGRAAPDTETDTGHTALIS
MSTRG.18017.1    ---------------SSALSVTS-------------------------------------
                                                                             


                        790       800       810       820       830       840
                 =========+=========+=========+=========+=========+=========+
Ec-12_003390     AVYRGQSSSVRSLIQEGADPNYSNRNGRTALMAAAAAGDPKMVIILLRHKADAARTDIHG
MSTRG.18017.1    -------------ITSGIE-----------------------------------------
                                                                             


                        850       860       870       880       890       900
                 =========+=========+=========+=========+=========+=========+
Ec-12_003390     KVAGAYAFERGFADLAQLLAIAAADGHEAAVDWELDRSRREEEEKERQRVQHLMDREGGV
MSTRG.18017.1    ------------------------------------------------------------
                                                                             


                        910       920       930       940       950       960
                 =========+=========+=========+=========+=========+=========+
Ec-12_003390     GGDGVPEADLYDWMIRVTKPREAAKIADRKLQSEQGSFVQAEPSKHNNNSSVSPPRGQQK
MSTRG.18017.1    ------------------------------------------------------------
                                                                             


                        970       980       990      1000      1010      1020
                 =========+=========+=========+=========+=========+=========+
Ec-12_003390     GRERCPKCTLFVPCLHFAGAKLPDGVAEWSVNQRGASRRANAGGKRLVYARSGNQSVAWW
MSTRG.18017.1    -----------------AGASVP------------ASRDSKAAAK---------------
                                                    ##########               


                       1030      1040
                 =========+=========+=========
Ec-12_003390     RTLHQAHRDRTLPPRPIERTRSSTPPVAD
MSTRG.18017.1    ---------------------------EQ
```

```
Parameters used
Minimum Number Of Sequences For A Conserved Position: 2
Minimum Number Of Sequences For A Flanking Position: 2
Maximum Number Of Contiguous Nonconserved Positions: 8
Minimum Length Of A Block: 10
Allowed Gap Positions: None
Use Similarity Matrices: Yes
```

```
Flank positions of the 3 selected block(s)
Flanks: [149  650]  [666  678]  [996  1005]  

New number of positions in Ec-12_003390.aa.fasta.trans.aa_ali.fasta-gb:  525  (50% of the original 1049 positions)
```
